# High-Resolution Shear Wave Imaging of the Human Cornea Using a Dual-Element Transducer

**DOI:** 10.3390/s18124244

**Published:** 2018-12-03

**Authors:** Pei-Yu Chen, Cho-Chiang Shih, Wei-Chen Lin, Teng Ma, Qifa Zhou, K. Kirk Shung, Chih-Chung Huang

**Affiliations:** 1 Department of Biomedical Engineering, National Cheng Kung University, Tainan 701, Taiwan; payyi.chen@gmail.com (P.-Y.C.); garyshihx@gmail.com (C.-C.S.); 2 Department of Microbiology and Immunology, National Cheng Kung University, Tainan 701, Taiwan; wcnikelin@mail.ncku.edu.tw; 3 Department of Biomedical Engineering, University of Southern California, Los Angeles, CA 90089, USA; teng.ma@siat.ac.cn (T.M.); qifazhou@usc.edu (Q.Z.); kkshung@usc.edu (K.K.S.); 4 Department of Ophthalmology, Roski Eye Institute, University of Southern California, Los Angeles, CA 90089, USA; 5 Medical Device Innovation Center, National Cheng Kung University, Tainan 701, Taiwan

**Keywords:** human corneal elasticity, high-resolution shear wave imaging (HR-SWI)

## Abstract

Estimating the corneal elasticity can provide valuable information for corneal pathologies and treatments. Ophthalmologic pathologies will invariably cause changes to the elasticity of the cornea. For example, keratoconus and the phototoxic effects of ultraviolet radiation usually increase the corneal elasticity. This makes a quantitative estimation of the elasticity of the human cornea important for ophthalmic diagnoses. The present study investigated the use of a proposed high-resolution shear wave imaging (HR-SWI) method based on a dual-element transducer (comprising an 8-MHz element for pushing and a 32-MHz element for imaging) for measuring the group shear wave velocity (GSWV) of the human cornea. An empirical Young’s modulus formula was used to accurately convert the GSWV to Young’s modulus. Four quantitative parameters, bias, resolution, contrast, and contrast-to-noise ratio (CNR), were measured in gelatin phantoms with two different concentrations (3% and 7%) to evaluate the performance of HR-SWI. The biases of gelatin phantoms (3% and 7%) were 5.88% and 0.78%, respectively. The contrast and CNR were 0.76, 1.31 and 3.22, 2.43 for the two-side and two-layer phantoms, respectively. The measured image resolutions of HR-SWI in the lateral and axial directions were 72 and 140 μm, respectively. The calculated phase SWV (PSWV) and their corresponding Young’s modulus from six human donors were 2.45 ± 0.48 m/s (1600 Hz) and 11.52 ± 7.81 kPa, respectively. All the experimental results validated the concept of HR-SWI and its ability for measuring the human corneal elasticity.

## 1. Introduction

The elastic properties of the cornea are crucial to human vision [1]. The corneal elasticity can be influenced by ophthalmic pathologies and surgical treatments. Ophthalmic pathologies such as keratoconus and the phototoxic effects of ultraviolet radiation cause the corneal elasticity change [2,3,4]. Corneal treatments such as laser-assisted in situ keratomileusis (LASIK) refractive surgery [5] and corneal collagen cross-linking treatment [6] are related with corneal elasticity. Therefore, measuring the elasticity distribution of the cornea is important for evaluating corneal pathologies and the efficacy of corneal treatment, particularly during the early stages of corneal sclerosis. Currently, the intra-ocular pressure (IOP) measurement is in routine use in ophthalmology. The most common method currently used to evaluate the IOP in ophthalmology is tonometry. An ocular response analyzer (ORA; Reichert Inc., Depew, NY, USA) that utilizes measurements of the IOP has been developed for evaluating the mechanical properties of the cornea in vivo [7]. However, the ORA cannot provide the local elasticity distribution within a small region of the cornea, which is especially important for the early detection of corneal sclerosis. Although the disadvantage may be overcome by characterizing the ultrasonic parameters of eye tissues, such as attenuation [8,9], velocity [10], and backscattering statistical parameters [11], a quantitative method for directly measuring the elasticity distribution of the cornea is still needed.

Elastography has been widely developed to measure the mechanical properties of soft tissues by using both ultrasonic and optical approaches. Optical coherence elastography (OCE) was utilized to detect the mechanically induced response of the tissue for providing an elasticity distribution map with high spatial resolution. However, some OCE methods use a tilted air-pulse [12,13,14] or a tilted air-coupled ultrasound transducer [15] to create a mechanical response in tissue, which cause a complex tissue response in the tissue and it may result in bias or error estimation [16]. Ultrasound-based elastography was first proposed by Ophir to measure the strain distribution of soft tissues based on applying an external force to the tissue and measured its deformation at different depths [17]. The measured internal strain profile along the probe direction can be used to reconstruct the strain distribution within the tissue. However, the elasticity distribution of small organs such as the cornea can only be detected if the image resolution is sufficiently high. High-frequency ultrasound (>30 MHz) imaging has a high sensitivity and resolution for detecting the microstructure and superficial tissues [18,19,20]. This has prompted researchers to use high-frequency ultrasound to measure the corneal elasticity. Hollman et al. used a 50-MHz elasticity microscope to measure the strain distribution of the normal porcine cornea [21]. Although that study provided high-frequency strain images that allowed various corneal layers to be distinguished, the method is not suitable for clinical patients due to the possibility of injuring the cornea.

This situation prompted the development of “remote palpation” for evaluating the biomechanical properties of the cornea. One such advanced approach based on using acoustic radiation force (ARF) to measure the mechanical properties of soft tissues has been developed [22]. This technique creates an ARF to remotely induce localized vibrations in the tissue, and the corresponding tissue dynamic response can be used to calculate the elastic properties. Several imaging modalities based on ARF for both qualitatively and quantitatively detecting the mechanical properties of soft tissues, such as vibroacoustic imaging [23], harmonic imaging [24], acoustic radiation-force-impulse (ARFI) imaging [25], and shear wave elasticity imaging [26], have been reported. A single-element focused ultrasound transducer was used to both generate an ARF on tissue and image the induced displacement for noninvasive evaluation of the cornea biomechanics changes induced by cross-linking therapy [27]. A novel concept of using a confocal transducer to reconstruct high-resolution ARFI (HR-ARFI) images for detecting localized corneal sclerosis was proposed in our laboratory [28]. However, measuring the corneal elasticity quantitatively using ARFI approaches remains difficult since the actual magnitude of the applied force is unknown due to many factors, such as the absorption and reflection effects of cornea. A quantitative method called supersonic shear imaging (SSI) was developed for evaluating the corneal elasticity based on measuring the group shear wave velocity (GSWV) within the tissue [29]. Although SSI can provide quantitative elasticity images, the relatively low operating frequency limited the image resolution. The highest available frequency of SSI was 15 MHz, the resolution of such frequency is inadequate for imaging the cornea. Ultrasound biomicroscopy imaging systems used to examine the anterior segment of the eye, including the cornea, typically operate at frequencies of 30–50 MHz, which is adequate for ophthalmology applications, particularly for corneal imaging [30]. Currently, no high-frequency SSI system is available, which prompted us to develop a high-resolution shear wave imaging (HR-SWI) method for measuring the corneal elasticity.

The purpose of this study was to develop an HR-SWI method for measuring the elasticity of the human cornea. A special dual-element transducer was designed in this study: the 8-MHz pushing element was excited to generate an ARF to induce the propagation of a shear wave (SW) in the tissue, while the 32-MHz imaging element was used to measure the GSWV in order to perform HR-SWI. Gelatin phantom experiments were performed in order to measure the performance of HR-SWI. Four quantitative parameters, bias, resolution, contrast, and contrast-to-noise (CNR), were measured. Finally, six human donor corneas were measured by HR-SWI. All of the obtained experimental results demonstrated the potential usefulness of HR-SWI for assessment of corneal elasticity.

## 2. Materials and Methods

### 2.1. Dual-Element Transducer

**Figure 1:**
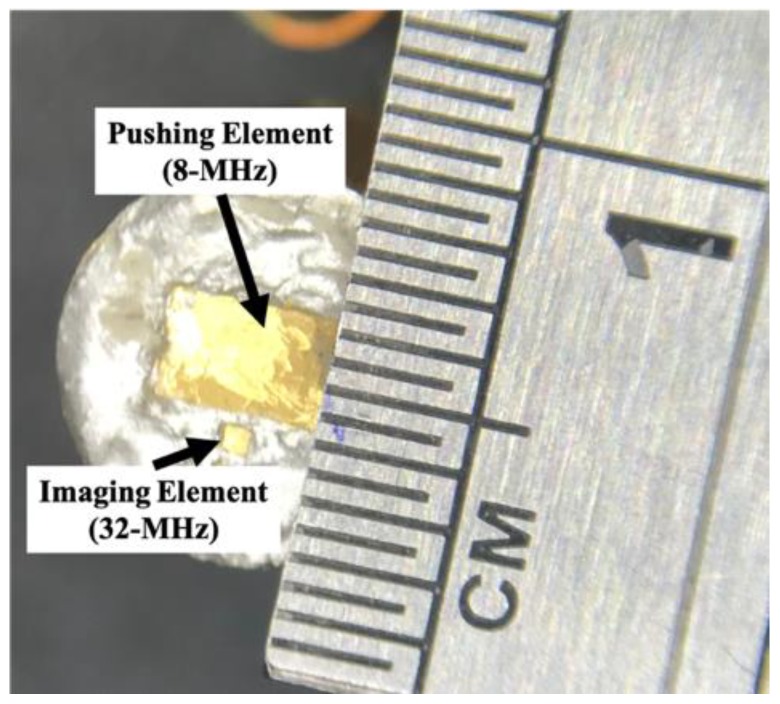
Photograph of the dual-element transducer. The low-frequency pushing element of 8-MHz was used to create a radiation force to induce a SW in tissues. The high-frequency imaging element was used to monitor the motion of the induced SW. The distance between the pushing and imaging elements is 1.5 mm.

Figure 1 shows the dual-element transducer fabricated in this study (NIH Ultrasonic Transducer Resource Center, University of Southern California, Los Angeles, CA, USA). An 8-MHz pushing element was used to create an ARF that induced the propagation of a SW in the tissue, while a 32-MHz imaging element was used to monitor the motion of the SW. The aperture sizes for pushing and imaging element are 2.02 × 2.61 mm and 0.55 × 0.56 mm, respectively. Two connectors were designed to reduce the electrical interference between signals at the two frequencies. The distance between the pushing and imaging beams (i.e., between the centers of the two elements) was 1.5 mm. The sample volume of imaging element was located at a depth about 1.2 mm for measurement. The acoustic output pressure of the pushing beam was measured using a calibrated hydrophone (NH0200, Precision Acoustic, Dorset, UK). When the transducer was excited by the transmitter at a peak-to-peak amplitude of 80 V, the peak acoustic pressure level was 0.37 MPa, the measured I_SPTA_ was 5.67 mW/cm^2^, and the mechanical index was 0.13. According to the FDA regulations for ophthalmic applications [31] (I_SPTA_ < 17 mW/cm^2^ and the MI < 0.23), the dual-element transducer complies with the regulations.

### 2.2. Experimental Setup for HR-SWI

**Figure 2:**
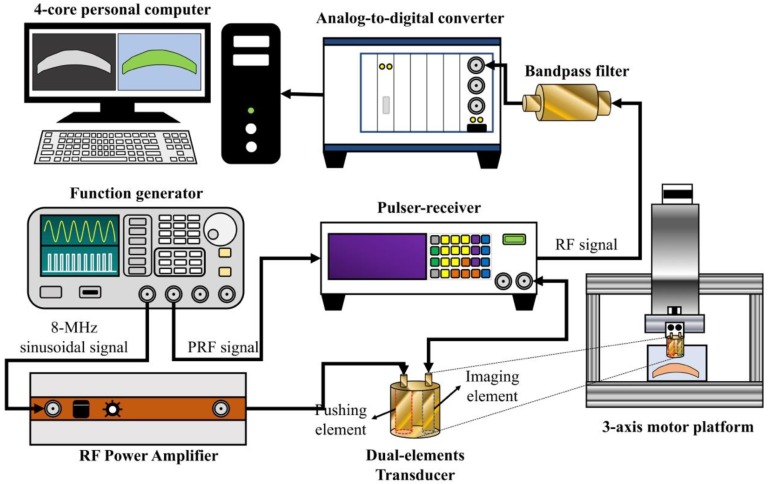
Experimental setup of the HR-SWI system. The function generator was used to generate the excitation signal for the pushing element and the trigger the imaging element. The 3-axis motor platform was used to move the dual-element transduce for measuring the GSWV at different locations.

Figure 2 shows the experimental setup for HR-SWI. A function generator (AFG3252, Tektronix, Beaverton, OR, USA) was connected to a radio-frequency (RF) power amplifier with a minimum output power of 25 watts (25A250, Amplifier Research, Souderton, PA, USA) that generated sinusoidal 8-MHz tone bursts of 0.5-ms duration to excite the pushing element of the dual-element transducer. The RF power amplifier produced an excitation voltage up to 80 V. A pulser-receiver (5900PR, Panametrics, Waltham, MA, USA) with a 200-MHz bandwidth excited the 32-MHz element of the dual-element transducer for transmitting and receiving the ultrasound-backscattering signal with a pulse repetition frequency (PRF) of 10 kHz. The ultrasound signals backscattered from the tissue were amplified 40 dB and filtered using a built-in variable-gain amplifier (variable gain from 26 to 50 dB) and bandpass filter (a five order Butterworth bandpass filter with a cutoff frequency from 32 to 48 MHz), respectively. The pulser-receiver was triggered by the function generator. The PRF trigger of the function generator was also used to synchronize the acquisition of the backscattered signals at a sampling frequency of 1 GHz by an 8-bit analog-to-digital converter (PXI-5152, National Instruments, Austin, TX, USA). After data acquisition, a personal computer with a four-core CPU was able to reconstruct the HR-SWI images within a minimum of 0.5 s. In order to repeat the data acquisition procedure, the internal trigger interval of the function generator was used to synchronize the whole system while the pushing and imaging sequences operated continuously.

### 2.3. Timing Diagram

**Figure 3:**
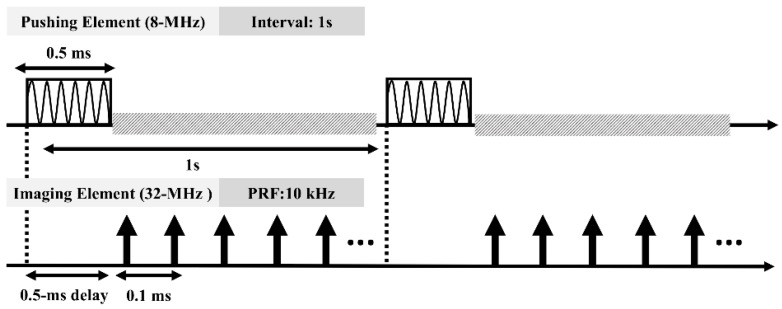
Timing diagram for the pushing and imaging sequences. A 500 µs tone burst was for the pushing elements to create s SW. To avoid the interference between pushing and imaging elements, the 500 µs delay was set before acquiring the data. The interval of 1s was used to synchronize the sequences of pushing and imaging elements.

Figure 3 illustrates the timing sequence for both elements. The excitation signal for the pushing element was coupled to the backscattering signal while generating a SW. Interference between the pushing and imaging sequences affects the accuracy of data acquisition, and this was avoided by introducing a 0.5-ms delay in the imaging sequence. The data acquisition procedure was triggered at an interval of 1 s, which is sufficient for the tissue to relax fully. In order to eliminate bias and jitter from the recorded displacements, the displacement was recorded by 500 A-lines at each location and a total number of data set is 10. In total, 5000 (500 × 10 = 5000) A-lines were recorded at each location for monitoring the tissue displacement.

### 2.4. Scanning Scheme

**Figure 4:**
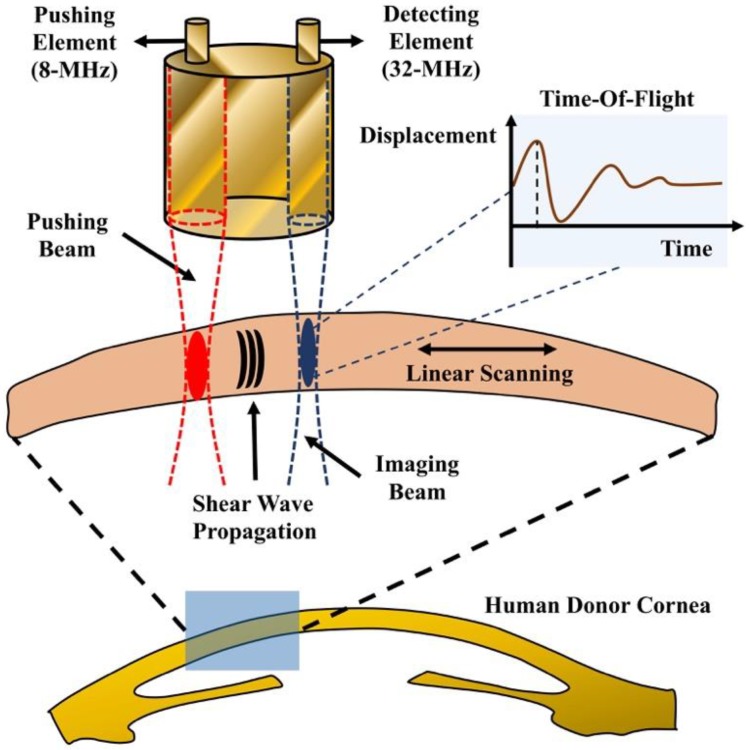
Timing diagram for the pushing and imaging sequences. A linear scanning was performed to measure the GSWV at different lateral locations.

Figure 4 shows a scanning diagram of HR-SWI. The dual-element transducer was attached to a three-axis motor platform to perform linear scanning with a lateral step size of 50 μm. The window size is 77 μm with 50% overlap. At each scanning location, the axial depth data length of the acquired ultrasound-backscattering signal was 1.7 mm for the phantom experiments and 1.54 mm for the cornea experiment. A time-of-flight (TOF) algorithm was used to calculate the GSWV at different locations [32]. Finally, a 2-D HR-SWI image can be reconstructed by the measured GSWVs at different depth. The signal and image reconstruction were conducted using MATLAB 2017b (MathWorks, Natick, MA, USA).

### 2.5. Lamb Wave Empirical Young’s Modulus Estimation

A Lamb wave empirical formula was used to derive the corneal elasticity from the measured GSWV. This formula has been widely used in superficial tissues such as cornea and arterial wall [29]. For a plate-like tissue, the phase SWV (PSWV) can be derived from the GSWV by using an empirical formula, which is based on the Lamb theory [29]:

(1) $$V_{\mathrm{phase}}=\sqrt{\frac{\omega \cdot h\cdot V_{\mathrm{group}}}{2\sqrt{3}}}$$

where $V_{\mathrm{group}}$ is the GSWV, $V_{\mathrm{phase}}$ is the PSWV, h is the thickness of cornea, and ω is the angular frequency of SW-induced tissue response. The Young’s modulus can be calculated by reformulating (1):

(2) $$E=36\frac{{\rho V}_{\mathrm{phase}}{}^{4}}{h^{2}\omega^{2}}$$

where E is Young’s modulus and ρ is tissue density.

### 2.6. Performace of HR-SWI

In order to evaluate the performance of HR-SWI, four quantitative parameters: resolution, bias, contrast, and contrast-to-noise ratio (CNR) were measured. The bias was used to compare the measured values of HR-SWI with the nominal value from other elastography methods [33]:

(3) $$\mathrm{Bias}=\frac{\left|V_{measured}-V_{nominal}\right|}{V_{nominal}}$$

where $V_{measured}$ is the measured GSWV from HR-WI and $V_{nominal}$ is the GSWV from other elastography techniques with the same gelatin concentration [33,34]. And the contrast and CNR were defined as follows [34]:

(4) $$\mathrm{contrast}=\frac{\left|V_{Layer1}-V_{Layer2}\right|}{V_{Layer2}}$$

(5) $$\mathrm{CNR}=\frac{\left|V_{Layer1}-V_{Layer2}\right|}{\left(\sigma_{Layer1}+\sigma_{Layer2}\right)/2}$$

where $V_{Layer1}$ is the GSWV of first layer, $V_{Layer2}$ is the GSWV of second layer, and $\sigma_{Layer1}$ and $\sigma_{Layer2}$ are the standard deviations of GSWVs of these two layers. The resolution is defined by the product of a sigmoid function [34]:

(6) $$V\left(x\right)=\left(V_{1}-V_{2}\left[\frac{1}{1+e^{-\frac{x-x_{1}}{\lambda }}}\right]\right)+V_{2}$$

where V(x) is the measured GSWV profile, x is the lateral or axial location, $V_{1}$ and $V_{2}$ are the measured GSWVs in the two layers, $x_{1}$ is the position of the boundary between the two layers, and λ is the width of the transition between the two layers. These four parameters can be obtained using the lsqcurvefit function in MATLAB. The image resolution can then be defined as the distance between the 20% and 80% transition of the GSWV profile [34]:

(7) $$R_{2080}=\frac{\sum_{i=1}^{N}2\ln\left(4\right)\left|\lambda_{i}\right|}{N_{Profiles}}$$

where $N_{Profiles}$ is the number of total GSWV profiles across the two layers.

### 2.7. Gelatin Phantom

The actual performance of the HR-SWI was experimentally verified by two types of gelatin phantoms. Two types of phantoms were constructed to two-side (left and right) and two-layer (top and bottom) tissue-mimicking phantoms from 300 Bloom gelatin powder (type A, Sigma-Aldrich, St. Louis, MO, USA) with two concentrations (3% and 7%) to simulate tissues with different stiffnesses. The graphite powder with a particle size smaller than 25 µm (Sigma-Aldrich) was added to gelatin phantoms with a concentration of 1% to provide the ultrasonic backscattering signal. A step-by-step procedure for the phantom fabrication was described here. First, the gelatin powder and water were prepared by weight. Gelatin powder was presoaked in the water for about 15 min while being stirred before heated to 80 °C. After the whole solution became optically transparent, the graphite powder was added and waited for the graphite powder well dissolved. Subsequently, the whole solution was cooled down to 30 °C and place in a phantom model and stored in a refrigerator for 2 h. Due to the stiffness of gelatin-based phantoms varying with temperature, all the experiments were performed at 25 °C. The number of samples for each type is four.

### 2.8. Human Corneal Samples

In this study, we used the outer edge of the donor corneal tissues which were removed from transplantation [35]. The cornea samples originated from healthy donors. The healthy corneas were initially used for transplant surgery, and the remaining parts of corneas were provided for the ultrasound experiments. Therefore, because only part of the corneas was available, an artificial anterior chamber was not suitable for fixing the cornea sample. Instead, each corneal sample was fixed on a container using 3% gelatin to allow the experiments to be performed.

## 3. Results

**Figure 5:**
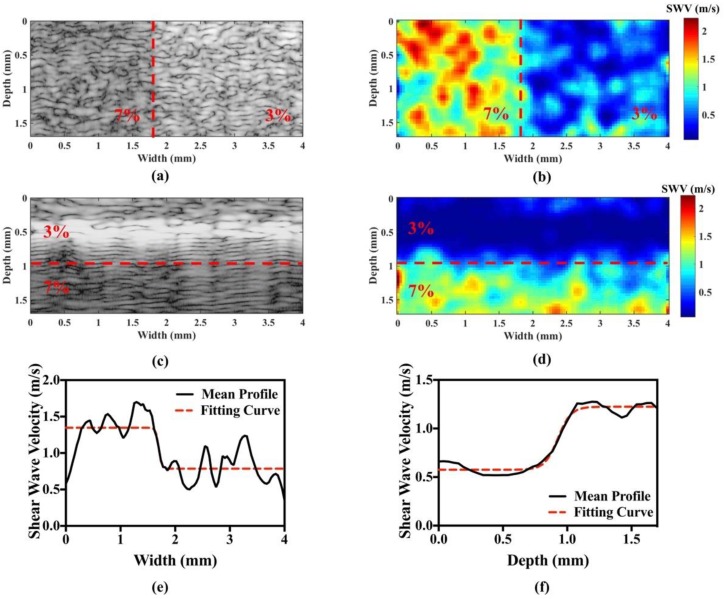
Results of phantom experiments. B-mode image (**a**) and its corresponding HR-SWI image (**b**) of a two-side (left and right) phantom. B-mode image (**c**) and its corresponding HR-SWI image (**d**) of a two-layer (top and bottom) phantom. Image resolution profile of HR-SWI for the axial (**e**) and lateral (**f**) directions.

Figure 5 shows B-mode images and their corresponding HR-SWI images for the homemade two-side and two-layer phantoms. For the two-side phantom, the width of the left side is 1.7 mm and 2.3 mm for the right side. For the two-layer phantom, the thickness of the top layer is 1 mm and 0.7 mm for the bottom layer. The image dimensions are 1.7 mm × 4 mm. As shown in Figure 5a, the boundaries between soft and hard stiffnesses in two-side phantom cannot be easily distinguish. But it can be clearly distinguished in the HR-SWI image, as shown in Figure 5b. The areas in red (corresponding to 7% gelatin) in the image indicate that they are stiffer than the blue areas (corresponding to 3% gelatin). The mean GSWVs in the left and right regions were 0.76 ± 0.13 (3% gelatin) and 1.34 ± 0.23 (7% gelatin) m/s, respectively. Similarly, the B-mode image and its corresponding HR-SWI image for the two-layer phantom are shown in Figure 5c,d, respectively. The difficulty in fabricating a two-layer phantom with such small dimensions can result in the deposition of the added graphite powder during gelatin solidification, leading to a brightness difference in the B-mode images for the two layers, as evident in Figure 5c. Nevertheless, the stiffness distribution within the phantom can be clearly distinguished in the HR-SWI image, as shown in Figure 5d. The mean GSWVs for the top (3% gelatin) and bottom (7% gelatin) layers were 0.52 ± 0.22 and 1.2 ± 0.34 m/s, respectively. The mean GSWV profiles in both the horizontal and vertical directions and their corresponding fitting curves are shown in Figure 5e,f. The estimated lateral and axial resolutions were 72 and 140 μm, respectively. The contrast and CNR for the two types of phantoms were 0.76, 1.31 and 3.22, 2.43, respectively.

**Figure 6:**
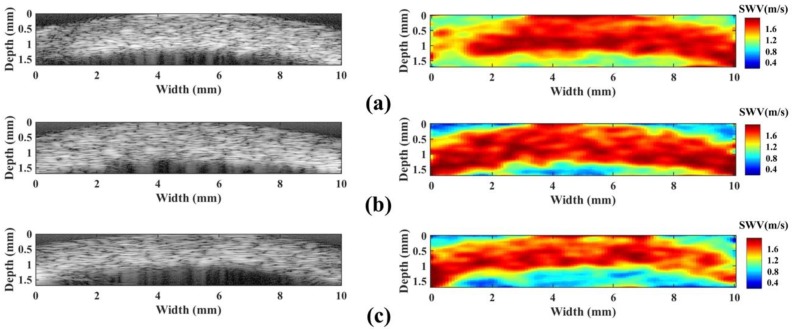
B-mode images and their corresponding HR-SWI images of the human corneas from three donors (**a**–**c**). Left: B-mode images. Right: SWI images.

Typical B-mode images and their corresponding HR-SWI images for three human corneas are shown in Figure 6. The dimensions of these images are 1.54 mm × 10 mm. The SWVs outside of corneal tissue region was the noise from TOF algorithm because the TOF algorithm cannot perform well in no tissue motion region. The structure of the cornea can be clearly seen in the B-mode images of all of the samples. However, the axial resolution is not very good in these images due to the imaging element having no focusing. The stiffness distributions of these corneas were observed in the HR-SWI images. The measured GSWV of the human cornea (*N* = 6) was 1.54 ± 0.43 m/s (mean ± SD). In this study, the central frequency of SW-induced response is 1600 Hz and thus the PSWV of 2.45 ± 0.48 m/s was calculated according to the GSWV.

## 4. Discussion

The use of ARF for SWI is the most common approach for obtaining the elasticity mapping of tissues and gradually becomes a basic function of current ultrasound imaging systems. The highest operational frequency for most clinical ultrasound system with the SWI ability is about 18 MHz. However, this frequency is inadequate for measuring the superficial tissues such as cornea. A frequency higher than 30 MHz is suitable for corneal applications. However, it is difficult to use a high frequency ultrasound array transducer to create an ARF for inducing a SW in tissues. Generation of ARF needs to deliver a high voltage with a long duration (typically a few hundred milliseconds) to the high-frequency transducer, which may directly destroy the high-frequency ultrasound elements. HR-SWI successfully utilized a novel concept of comprising the elements with two different frequencies to overcome this problem. The element of 8 MHz was used to create an ARF to induce a SW and the high-frequency element of 32 MHz was used to measure the SW. Then the HR-SWI images can be obtained by applying a linear scanning. 

The performance of HR-SWI was quantified by four parameters: bias, resolution, contrast, and CNR. The bias of SWV is important for assessing the tissue elasticity. Compared to other elastography techniques [36,37], the biases of HR-SWI for 3% and 7% gelatin phantom were 5.88% and 0.78%, respectively. These values are in an acceptable range (<6%). The effects of contrast and CNR have been reported as a limiting factor for elastography. A previous study measured the contrast and CNR for an inclusion phantom using TOF-based methods [38]. The contrast ranged from 0.15 to 1.44 and the values of CNR were from 0.6 to 6.23. In the present study, the reconstruction algorithm of HR-SWI also depends on the TOF algorithm. Therefore, the contrast of 0.76 (two-side phantom) and 1.31 (two-layer-phantom) and the CNR of 3.22 (two-side phantom) and 2.43 (two-layer-phantom) are in a reasonable range. Moreover, the corneal elasticity for the abnormal cornea is around 2 kPa, and 4 kPa for the healthy cornea [39]. An approximation of contrast based on (4) between the abnormal and healthy corneas is around 0.5. The contrast of HR-SWI are higher this value, which demonstrates its ability to distinguish the difference between abnormal and healthy corneas. Except for the contrast and CNR, the resolution is another important parameter for a SWI image. An interesting finding is that the lateral resolution of HR-SWI is smaller than the axial resolution, this trend is opposite to the typical ultrasound imaging. In typical ultrasound imaging, the axial and lateral resolutions main depend on the pulse length, wavelength, and f-numbers of the ultrasound transducer. However, in ultrasound elastography, the axial resolution is defined by the pulse length and the window size that used in the image reconstruction algorithm. On the other hand, the lateral resolution depends on the imaging element. Due to the need of mechanical scanning for SWI, the lateral resolution of HR-SWI depended on the lateral step size of the scanning system. This may be used to explained the image resolution trends difference in these two image techniques. 

In our previous study, we reconstructed HR-ARFI images of the porcine cornea obtained using a dual-frequency transducer comprising elements operating at 11 and 48 MHz [28]. The dual-frequency transducer was designed to be confocal at the same depth, and it provided image resolutions of 153 and 177 μm [28]. HR-ARFI is currently the ARF-based method that provides images of corneal elasticity with the highest resolution. The measured lateral and axial resolutions of HR-SWI were 72 and 140 μm, which is better than HR-ARFI. This seems to in a good agreement with the previous study [40], which compared the performance between ARFI and SWI images, and the results showed that the SWI image outperformed ARFI in terms of resolution. Moreover, the resolution is also affected by the elasticity difference between two layers/sides medium according to (6). The elasticity difference between healthy and abnormal corneas is around twice [39], which is similar to the elasticity difference in the phantom experiment in the present study. Therefore, the measured image resolution of HR-SWI is close to the real situation for differentiating the abnormal and healthy corneas.

In addition, HR-ARFI imaging needs mechanical scanning to be performed at multiple axial depths (brightness/depth [B/D] scan) to ensure that the region of interest is located in the focal zone. B/D is a special mechanical scanning method that moves the transducer in both depth and lateral directions to acquire data. This requirement results in a typical data acquisition time of around 50 min [28]. Due to the thickness of the human cornea, the applied ARF can induce sufficient tissue displacement for the penetration of SWs to be detected by the imaging element. Therefore, the imaging sequence of HR-SWI can be performed without a B/D scan, which significantly reduces the data acquisition time. Even though HR-SWI still requires mechanical scanning, the minimum data acquisition time is reduced to about 10 s. The abilities of fast scanning and providing a quantitative elasticity information make HR-SWI more suitable for corneal applications.

The cornea is considered to be similar to a thin plate, which is surrounded on one side by the aqueous humor and on the other side by a coupling liquid/water during the ultrasonic investigation. A strong contrast in the SWV exists between the cornea and its surrounding liquids. When SWs propagates in cornea, the strong reflection and mode conversions partially guide the propagated waves, resulting in a so-called Lamb wave. Lamb waves propagate with multiple modes, and each mode has its own highly dispersive curve. The relation between Young’s modulus and GSWV is more complex than the well-known E = 3*ρ*c^2^ formula. Measurement of the PSWV dispersion curve of cornea is essential to assess the Young’s modulus of cornea [12,13,41,42]. However, the design of the dual-element transducer can only measure the GSWV of tissue. Fortunately, this limitation can be overcome by using an empirical Young’s modulus formula based on the GSWV [29], as described in (2). In the present study, the central frequency of the SW induced tissue response, the thickness, and the GSWV were 1600 Hz, 1.35 ± 0.15 mm, and 1.54 ± 0.43 m/s, respectively, which is corresponding to the PSWV of 2.45 ± 0.48 m/s. The corresponding Young’s modulus of cornea is 11.52 ± 7.81 kPa. The elasticity of the human cornea has been intensively investigated using different methodologies [43,44,45,46,47], as shown in Table 1. The corneal elasticity is in a wide range from 0.5 to 269.01 kPa. However, the physiological conditions of corneal samples from literature is different, which may also change the corneal elasticity. For example, the elastic modulus has been experimentally found to increase linearly with the IOP [48], while it also increases with age [49]. In the present study, the cornea samples originated from healthy donors. The healthy corneas were initially used for transplant surgery, and the remaining peripheral parts of corneas were provided for the ultrasound experiments. In this study, we were unable to control the physiological conditions of cornea samples such as age or IOP for complete comparison with literatures. A rough comparison between literature and HR-SWI shows that the values from HR-SWI seem to be reasonable. This can be used to validate the concept of using a dual-element transducer for measuring the shear wave images of the cornea. Due to measuring the corneal elasticity under IOP is more meaningful, future works will focus on applying HR-SWI for measuring the human corneal elasticity under IOP condition.

There were some limitations in the present study. (1) The distance between the two elements are quite close to each other, which results in an interference between these two elements during SWI and may reduce the signal-to-noise ratio of SWI images. A special transducer fabrication design should be developed to isolate the pushing and imaging elements. (2) The human samples were obtained after cornea transplant surgery, therefore, the corneal shape in this study is not fully the same to a real situation. But the concept of HR-SWI for human sample applications was proved in this study. The effect of IOP should be considered in the next works. 

## 5. Conclusions

The present study has demonstrated the feasibility of using HR-SWI for measuring the elasticity of the human cornea. A dual-element transducer was designed to generate the ARF (using the 8-MHz pushing element) to induce a propagating SW that can be simultaneously monitored using the 32-MHz imaging element. Because HR-SWI only measured the GSWV of tissues, an empirical Young’s modulus formula was used to convert the GSWV to Young’s modulus to solve the Lamb wave problem in corneal tissues. In phantom experiments, HR-SWI could quantitatively distinguish two-side phantoms with dimensions of 1.7 mm × 4 mm, and the real image resolutions in the lateral and axial directions were 72 and 140 μm, respectively. The mean GSWVs for 3%- and 7%-gelatin phantoms were 0.64 and 1.27 m/s, respectively. The bias of HR-SWI for phantoms is less than 6%. The SWI of cornea from six human donors were obtained. The measured PSWV of the human cornea was 2.45 ± 0.48 m/s at the SW frequency of 1600 Hz, which corresponds to Young’s modulus of 11.52 ± 7.81 kPa (*N* = 6). All of the obtained experimental results demonstrate that using the dual-element transducer to perform HR-SWI reconstruction has great potential for clinical applications in ophthalmology.

## Figures and Tables

**Table 1:** Results of studies measuring the human corneal elasticity.

Methodology	Shear Modulus (kPa)	IOP (mmHg)	Reference
Inflation test	^2^ 55.6–123.9	No	[40]
Atomic force microscopy	^2^ 1.79–27.39	No	[41]
Nanoindentation	10.21–269.01	No	[42]
^1^ ARFEM	0.5–1.67	20	[43]
Needle indentation	0.75–1.75	50	[44]
The present study	3.71–19.33	No	

^1^ Acoustic radiation force elasticity microscopy. ^2^ Original data were given by Young’s modulus (E), shear modulus (G) here is calculated by the formula of G = E/2(1 + ν), where ν is Poisson ratio of 0.42 [46].
